# Machine learning approaches for parsing comorbidity/heterogeneity in antisociality and substance use disorders: A primer

**DOI:** 10.1017/pen.2021.2

**Published:** 2021-11-15

**Authors:** Matthew S. Shane, William J. Denomme

**Affiliations:** Ontario Tech University, Forensic Psychology, Oshawa, ON, Canada

**Keywords:** Externalizing, Antisocial, Substance abuse, Neuroimaging, Machine learning

## Abstract

By some accounts, as many as 93% of individuals diagnosed with antisocial personality disorder (ASPD) or psychopathy also meet criteria for some form of substance use disorder (SUD). This high level of comorbidity, combined with an overlapping biopsychosocial profile, and potentially interacting features, has made it difficult to delineate the shared/unique characteristics of each disorder. Moreover, while rarely acknowledged, both SUD and antisociality exist as highly heterogeneous disorders in need of more targeted parcellation. While emerging data-driven nosology for psychiatric disorders (e.g., Research Domain Criteria (RDoC), Hierarchical Taxonomy of Psychopathology (HiTOP)) offers the opportunity for a more systematic delineation of the externalizing spectrum, the interrogation of large, complex neuroimaging-based datasets may require data-driven approaches that are not yet widely employed in psychiatric neuroscience. With this in mind, the proposed article sets out to provide an introduction into machine learning methods for neuroimaging that can help parse comorbid, heterogeneous externalizing samples. The modest machine learning work conducted to date within the externalizing domain demonstrates the potential utility of the approach but remains highly nascent. Within the paper, we make suggestions for how future work can make use of machine learning methods, in combination with emerging psychiatric nosology systems, to further diagnostic and etiological understandings of the externalizing spectrum. Finally, we briefly consider some challenges that will need to be overcome to encourage further progress in the field.

Imagine that a 42-year-old man, diagnosed with antisocial personality disorder (ASPD), psychopathy, and a substance use disorder (SUD), is administered a series of structural, functional, and resting-state brain scans. The results of the scans highlight several potentially important features: reduced hippocampal volume, reduced activity within the anterior cingulate, and reduced connectivity within the default mode network. But what can we conclude from these findings? Do they underlie all of this man’s diagnoses? Or just one of them? And if only one – which one? Moreover, do they represent endogenous features of the man’s neural environment, which may serve as biomarkers, or as predisposing factors, for these disorders? Or do they instead merely represent the consequences of a lifetime of antisocial behavior and/or substance abuse? The empirical literature can support either possibility: those at risk for externalizing disorders do show unique neural features that appear to predate their problematic behavior (Nguyen-Louie et al., 2018); likewise, participation in an antisocial lifestyle (Shepherd et al., 2009), or in long-term drug use (Volkow et al., 1992), can have clear, detrimental effects on brain health. However, because as many as 93% of individuals diagnosed with antisocial personalities also meet criteria for a SUD (Messina et al., 2000; Smith & Newman, 1990), and because these externalizing constructs are themselves highly heterogeneous (Fanti & Kimonis, 2017), delineation of the shared/unique features of each disorder has been difficult to achieve. Indeed, despite promises of improved diagnostics and precision psychiatry (e.g., Costafreda et al., 2009; Wium-Andersen et al., 2017), modern clinical neuroimaging has to date been unable to fully unravel the complexity of these and other important questions.

The reasons for these challenges are complex, but are believed to include at least two somewhat related factors. First, while a major goal of clinical neuroimaging has been to use the insights from brain-based inquiries to improve the validity of clinical diagnostic systems, most work to date has used the existing diagnostic categories (i.e., from DSM5 and ICD-10) as the “gold standard” and has referenced their neural findings against these categories. Second, until recently, both sample sizes and computational techniques have been challenged to fully dissect the finer-grained comorbidity and heterogeneity issues inherent in most psychopathology. With regard to externalizing disorders, while considerable work has separately evaluated the neural systems relevant to antisociality and addiction, little neuroimaging work has yet attempted to model the comorbidity between the two (but see Steele et al., 2018), or to extract shared/unique variance in neural outcomes/predictors (but see Abram et al., 2015; Denomme et al., 2018; Hyatt et al., 2019). Moreover, despite inherent diagnostic heterogeneity (Brazil et al., 2018; Kotov et al., 2017), most neuroimaging work to date has treated antisocial personalities and SUDs as homogenous groups. This is unfortunate, because it may be through a more detailed understanding of each disorder that a personalized process for the assessment, diagnosis, and treatment of psychiatric disorders will be realized.

Fortunately, solutions for these problems are presenting themselves. First, alternate classification systems, which allow psychopathology to be viewed as dimensional, rather than categorical (e.g., RDoC, HiTOP), are providing an improved structure through which conceptualization of neuropsychiatric disorders can develop (Kotov et al., 2017). Second, a variety of data-driven analysis techniques, including those incorporating machine learning techniques, are becoming increasingly employed to undertake large-scale evaluations of complex psychiatric/neuroscientific relationships (Dwyer et al., 2018; Janssen et al., 2018). In combination with larger datasets and multisite/consortium efforts, these developments are providing methods for deeply interrogating neural systems underlying psychopathology for comorbid relationships, diagnostic heterogeneity, and/or symptom interactions at the neuroscientific and/or phenotypic level (see Bzdok & Meyer-Lindenberg, 2018; Rutledge et al., 2019). Work employing these techniques is becoming more common in certain domains (e.g., Alzheimer’s: Liu et al., 2014; Moradi et al., 2015; dementia: Mathotaarachchi et al., 2017; Pellegrini et al., 2018; anxiety/depression: e.g., Hilbert et al., 2017), but has only recently begun to percolate through to other psychiatric disorders. Work focused on the externalizing spectrum remains highly nascent.

One reason for this is that personality theory, clinical psychopathology, neuroimaging, and machine learning require highly diverse forms of expertise. With this in mind, the present paper seeks to provide suggestions regarding how data-driven machine learning techniques can be productively merged with dimensional conceptualizations of psychopathology, toward a more comprehensive understanding of the neural systems underlying externalizing disorders. Several recent papers have provided explanations/tutorials regarding the use of these techniques for psychiatric neuroscience in general (Bzdok & Meyer-Lindenberg, 2018; Cearns et al., 2019; Durstewitz et al., 2019; Janssen et al., 2018; Rutledge et al., 2019; Woo et al., 2017). Our intent is not to repeat these tutorials, but rather to specifically highlight the potential synergy between these data-driven techniques and developing dimensional nosology for externalizing disorders. We begin by reviewing traditional categorical classification systems and briefly highlighting some of the benefits of emerging dimensional classification systems. Next, we discuss ways through which data-driven machine learning approaches may intersect with these developing nosologies to help drive future insights into both diagnostic and etiological understanding of the externalizing spectrum. We follow this with a review of the modest machine learning work conducted within the externalizing domain to date, and point out areas of strength and weakness within this nascent field. Finally, we provide suggestions for the field, and consider several challenges that will need to be overcome as future work is undertaken.

## 1. Categorical Versus Dimensional Classification of Externalizing

In line with the DSM5’s broader classification strategy, Section II defines ASPD and SUD as categorically distinct conditions, each defined by the extent to which an individual meets required criteria. For ASPD, an individual must show significant impairments in both self and interpersonal functioning, as well as antagonistic (e.g., manipulativeness, callousness) and disinhibitory (e.g., irresponsibility, impulsivity, risk-taking) personality traits. For SUD, diagnosis requires the individual to meet at least 2 out of the 11 criteria within a given 12-month period, including metrics of substance-related physiological reactivity (e.g., withdrawal, tolerance, craving), and/or behavioural and interpersonal consequences of substance use (e.g., social/interpersonal problems; hazardous/irresponsible use). While psychopathy is not officially included within the DSM5, assessment via its most common assessment instrument (the Psychopathy Checklist – Revised; PCL-R, Hare, 1991) has also classically been categorical: individuals score 0, 1, or 2 on each of the 20 criteria (e.g., grandiosity, manipulativeness, lack of empathy, irresponsibility, impulsivity, delinquency), and are assessed as psychopathic via any combination of scoring that reaches at least 30 (out of the possible total of 40).

These classification systems have a long history, and have been seminal in enhancing the reliability of clinical/forensic diagnoses (though see Regier et al., 2013). Moreover, they have stimulated several generations of research into the etiology of psychopathology, and have helped generate the majority of therapeutic regimens in place today. Nonetheless, there are some significant limitations to these categorical approaches that impact their validity (see Insel et al., 2010; Widiger, 1992), and lessen their usefulness for scientific inquiry (see Hopwood et al., 2015; Krueger et al., 2014). First, separation of these externalizing disorders along arbitrary clinical lines hinders the ability to fully investigate known issues of comorbidity and symptom overlap. For instance, several symptoms show common overlap across all externalizing disorders (e.g., social/interpersonal issues, irresponsibility/risk-taking; Krueger et al., 2002; 2007), suggesting a potentially shared etiological basis. However, the ability to parse disorder-specific variance in symptom presentation, or to evaluate for comorbid interaction effects, is challenging because DSM-based comorbidity levels are so high (Smith & Newman, 1990).

Second, categorical approaches largely fail to acknowledge known heterogeneity within disorder categories. Both psychopathy and SUD (and to a lesser extent ASPD) can be diagnosed based on highly nonoverlapping sets of symptoms, which all but guarantee considerable within-category differences in symptom characteristics. Psychopathy, for instance, may be diagnosed with or without the presence of core affective or criminogenic traits (Skeem et al., 2007; Yildirim & Derksen, 2015). Similarly, SUD may be diagnosed with or without the presence of physiological withdrawal symptoms (Denomme et al., 2018; Schuckit et al., 2003). These differences may correspond to further heterogeneity in underlying causal mechanisms (e.g., see Fanti & Kimonis, 2017), or in the need for individualized treatment responses (see Baskin-Sommers et al., 2015). However, these important distinctions are difficult to identify when diverse individuals are placed within single diagnostic categories (Brazil et al., 2018).

Third, while both psychopathy and DSM5 SUD diagnoses (but not ASPD) do afford some rudimentary consideration of severity, by and large the all-or-nothing nature of existing classification systems affords little ability to separate mild from severe forms of the disorder. All told, these limitations impose downstream limits on scientific work focused on delineating underlying disorder mechanisms (e.g., cognitive, neural, genetic), and on progress toward truly individualized treatment protocols.

In acknowledgment of these weaknesses, concerted efforts have been placed on developing frameworks that conceptualize functional/dysfunctional states as dimensional, rather than categorical, phenomena. The National Institute of Mental Health has established their Research Domain Criteria (RDoC; Insel et al., 2010), which aim to encourage a bottom-up consideration of the basic building blocks of human functioning; while the Hierarchical Taxonomy of Psychopathology (HiTOP; Kotov et al., 2017) has used an extensive factor-analytic literature to demonstrate a hierarchical structure of psychopathology that can aid the development of an evidence-based dimensional nosology for mental disorders (similarly, the “triarchic model” of psychopathy has extended an empirically derived dimensional approach to the assessment of psychopathic characteristics (e.g., Patrick et al., 2009). Differences between these approaches are important (for instance, RDoC is a research system that makes no claims (as of yet) to providing formal diagnostic sets, whereas HiTOP is very much aimed at reforming diagnostic systems). However, the key to both efforts is the development of an increasingly dimensional hierarchy, which aims to distance psychiatric diagnosis from mere clinical intuition, and to instead base assessments in empirical patterns of psychological symptom co-occurrence (Conway et al., 2019; Cuthbert & Insel, 2013; Kotov et al., 2017).

For instance, HiTOP currently uses a six-level hierarchy to conceptualize various psychopathological states, the highest level serving as a placeholder for characteristics common across all psychopathological conditions (e.g., a “p factor”; Caspi et al., 2014), and the lowest level serving to differentiate individual presentations of clinical signs and symptoms (which may be unique to a specific disorder, or common to multiple disorders in varying degrees). Between these anchors, psychopathologies are distinguished by their empirical relationships, with natural variation in underlying processes serving as building blocks for higher-order latent factors. HiTOP does not utilize traditional diagnostic categories per se, but does include these categories within various formulations for a convenient link back to these “historical” diagnoses. For instance, with regard to externalizing disorders, HiTOP currently conceptualizes two higher-order spectra - disinhibited/antagonistic externalizing - which themselves may relate in differing degrees to substance-abusing and antisocial behaviors (e.g., SUDs vs. ASPD vs. psychopathy vs. other personality disorders), and to specific syndromes/disorders (e.g., Narcissistic/Histrionic/Paranoid/Borderline), respectively. These constructs and relationships are not written in stone, but rather are intended to serve as a set of malleable, testable hypotheses, which will adjust dynamically to continually mirror the empirically-derived literature.

Some valid critiques of these data-driven approaches have been tabled (e.g., Reed, 2018). However, there appears to be a considerable appetite for considering psychopathological states through the lens of these emerging nosologies. Doing so may not only improve clinical diagnostics and increase scientific utility, but may also afford modelling of disorder comorbidity (by allowing single lower-level features to load differentially on multiple higher-level syndromes), and heterogeneity (by allowing multiple lower-level features to load differentially on single higher-level syndromes (Conway et al., 2019; Cuthbert & Insel, 2013; Insel et al., 2010; Kotov et al., 2017; Latzman et al., 2020)). For instance, psychopathic individuals who do or do not present with core emotional deficits (see Schmitt & Newman, 1999) can be modelled independently (see Brazil et al., 2018); or multiple distinct neurobiological risk factors can each serve as independent risk factors for psychopathy (see Brislin & Patrick, 2019; Patrick et al., 2009), or SUD (see Koob & Volkow, 2010; Volkow et al., 2016). Such detailed individualization can in turn allow for better evaluation of underlying mechanisms, and for the development of more individualized treatment interventions.

## 2. Hypothesis-Driven Versus Data-Driven Analytic Approaches

Despite the renewed focus on psychopathological states as dimensional constructs, the majority of translational neuroimaging work on externalizing characteristics continues to use existing clinical categories as organizing features (i.e., DSM5/ICD-10). There may be several reasons for this, but one is the simple computational challenge inherent in analyzing neuroimaging data: most translational neuroimaging work utilizes a standard parametric mapping approach, wherein independent *t*-tests are used to test for changes (in either brain structure or function) at each of ˜50 000 voxels. Subsequently, to control for ever-present concerns regarding family-wise error rates, a combination of voxel- and cluster-based thresholding procedures are employed to identify significant, reproducible effects. While these methods have been productive overall, they tend to be limited to the interrogation of relatively streamlined analysis models – for instance, hypothesis-driven models that test for linear differences at each voxel. Analyses of this nature are good for testing hypotheses about specific brain–behavior relationships, or specific group-related differences. However, mapping complex multivariate symptom patterns onto similarly complex multivariate brain patterns is not something these analyses were designed to excel at (see Walter et al., 2019).

In recent years, the implementation of increasingly efficient data-driven techniques has made it possible to identify patterns in neuroimaging data structures with increased power and efficiency. In contrast to standard hypothesis testing, wherein a specific *a priori* hypothesis is evaluated against the null, these data-driven techniques can scour an entire dataset for statistical relationships in a partially (in the case of “supervised” techniques) or completely (in the case of “unsupervised” techniques) model-free manner (see below for definitions of supervised/unsupervised techniques, or for a comprehensive review within the context of psychiatric neuroscience, see Bzdok & Meyer-Lindenberg, 2018). While classically trained experimentalists will sometimes scoff at this “benefit”, by simultaneously evaluating the entire data structure for macro-level patterns, these techniques offer many advantages over traditional analysis techniques, including significantly heightened signal/noise ratios (Bzdok & loannidis, 2019), reduced need to control for multiple comparisons (Paulus et al., 2019), and an ability to incorporate more complicated multimodal (Sui et al., 2011) and latent factor (Bzdok & Meyer-Lindenberg, 2019) approaches. Moreover, by allowing findings to reflect the natural structure of the data, rather than presupposed hypotheses regarding symptom disorder clusters, researchers can reduce (though not completely remove; see Paulus et al., 2019; Woo et al., 2017) susceptibility to experimenter bias, and open new avenues for valuable insights (Huys et al., 2016). For instance, rather than *a priori* separating phenotypic symptoms along pre-defined lines, data-driven techniques can allow natural variation in the data to encourage novel clustering of characteristics (Bzdok et al., 2019; Walter et al., 2019). In the context of externalizing disorders, a data-driven approach may encourage a disruption of traditional diagnostic (i.e., DSM5) and neuroimaging (i.e., modular/ROI analyses) methods, and afford a more bottom-up, data-driven reconceptualization of symptoms/predictors according to empirically-derived variation in psychometric/biometric features. The convergence of these benefits, in combination with the capacity for more complicated multimodal/hierarchical models, makes them a logical fit for interrogating nuanced issues of comorbidity and heterogeneity.

Unfortunately, implementation of these data-driven techniques has historically required quite unique expertise, and utilization of these methods in the broad field of personality/psychiatric neuroscience (and the narrower study of externalizing disorders) remains under-represented. Moreover, the majority of work that has been undertaken to date has failed to take full advantage of the power that these machine learning techniques can offer. With this in mind, we describe below several of the more common forms that these data-driven pipelines can take, and provide several specific use cases through which each may have the capacity to facilitate our understanding of the shared/unique variance associated with externalizing symptoms/disorders. Following this, we review the modest body of externalizing work that has employed machine learning methods to date and use this work to highlight potential avenues for future inquiry.

## 3. Supervised Versus Unsupervised Approaches

Data-driven approaches can take two general forms: supervised and unsupervised. Supervised techniques make no assumptions about the data structure or the features to be extracted (i.e., the “independent variables”), but do require that the target being predicted (i.e., the “dependent variable”) be user-specified. Supervised techniques have thus become quite popular in psychiatric neuroscience for identifying neural patterns that maximally differentiate patient from control populations. Indeed, supervised pipelines have been used to identify neurobiological features that differentiate healthy controls from a wide variety of patient populations, including patients with schizophrenia (Shen et al., 2010), depression (Zeng et al., 2012), anxiety (Liu et al., 2015), autism (Bi et al., 2018), and Parkinson’s disease (Tang et al., 2017). Support Vector Machines (SVMs) are perhaps the most popular architecture used for these purposes, in part because of their ability to remain robust when employed on small samples (Melgani & Bruzzone, 2004), and in part because of their straightforward differentiation of target groups based on data features that create the most discriminant hyperplane (i.e., the “support vector”; Alpaydin, 2014). While there is no guarantee that the features identified by these data-driven techniques will ultimately have clinical/theoretical relevance, their mere ability to identify features that differentiate patient populations offers potentially important opportunities to improve diagnostic prediction and assessment. However, because the category labels (e.g., DSM5 disease categories) must be defined *a priori*, most current implementations of supervised models limit their opportunity to motivate truly transformational insights.

That said, there are ways in which supervised models can provide deeper insights into the nature of psychiatric neuroscience in general, and externalizing disorders in particular. For instance, just as supervised models can be used to differentiate patient from control groups, they can also be used to differentiate two or more patient groups. Thus, supervised models can aid differential diagnoses by requesting features that maximally differentiate disorders with known comorbidity or symptom overlap. This has been undertaken with some success in parallel fields (e.g., Du et al., 2015; Hilbert et al., 2017; Rashid et al., 2016), but has seen no uptake for differentiating antisocial personality features from SUDs to date. Alternately, predictive models could be designed to help delineate within-disorder heterogeneity by requesting features that maximally distinguish hypothesized within-disorder subtypes (e.g., relapse/abstinence, greater/lesser severity; Fede et al., 2019; Wetherill et al., 2018). As reviewed later in the paper, a number of studies have used techniques of this nature to begin differentiating based on prognosis or treatment success for psychopathy (Steele et al., 2018), and particularly for SUD (Bertocci et al., 2017; MacNiven et al., 2018); however, much less work has used supervised strategies to distinguish differential symptoms or disorder subtypes. Finally, with slightly adjusted data-driven architecture, supervised models can also be used to test continuous dimensions. For instance, Support Vector Regression (SVR) uses techniques similar to SVM, but is designed to identify a best-fit line that can predict variation in continuous metrics. This may make SVR particularly powerful for melding with dimensional nosology for externalizing disorders, which inherently view psychopathological states as existing on a continuum. Relevant topics for SVR may include the extent to which neural abnormalities track with lifetime substance use burden versus antisocial personality traits (see a similar approach by Denomme et al., 2018); the extent to which time in incarceration serves as an important factor for development or maintenance of different externalizing disorders; or the extent to which specific symptoms (e.g., impulsivity) may relate similarly or uniquely to different externalizing disorders. As with categorical SVM analyses, there remains no guarantee of clinical utility. However, when used in a responsible, data-driven pipeline that includes critical cross-validation strategies (see below for more discussions of this), confidence in the clinical relevance of extracted features/models can be increased considerably.

Unsupervised techniques, in contrast, make no assumptions about the data structure, the features being extracted, or the targets being predicted. The process thus becomes entirely data-driven, with the goal being only to lift out any patterns that emerge reliably from the data structure. As several well-known examples: a) development of the Big Five personality traits benefited greatly from the use of factor analysis, an unsupervised technique that uses blind source categorization to cluster variables based only on their maximum common variance (Goldberg, 1990); and b) identification of the brain’s resting-state networks has been greatly facilitated by the use of Independent Component Analysis (ICA; Allen et al., 2011; Beckmann et al., 2005), which has shown considerable utility for the identification and decomposition of statistically independent brain networks based on the grouping of spatially- and/or temporally coherent neural regions (Calhoun et al., 2008). Note that in both of these examples, there was little existing theory available to support specific factor/component structures. Indeed, this is where unsupervised models excel: in identifying statistical patterns that can serve as the basis for theoretical development. To place the utility of this approach in some context, consider an example: a researcher has a group of offenders and wants to evaluate the extent to which variation in neuroimaging features can provide novel information regarding the heterogeneity of the offender population. While a supervised model can be used to identify biomarkers that *confirm* existing offender categories, an unsupervised model can evaluate the natural variation in the underlying neural patterns and use that variation to *generate* new categories that may help explain previously unknown features of the population. Thus, when used within a broader pipeline that includes cross-validation procedures, unsupervised models may be viewed as particularly powerful in the early stages of theory building (in contrast to supervised models, which excel at theory confirmation/replication/extension). A full exposition of these ideas is well beyond the scope of this article, but interested readers may read Maia et al. (2017) for more thoughts regarding the use of data-driven pipelines for theoretical/atheoretical purposes.

### 3.1. A note on the importance of model validation

Perhaps the biggest criticism of data-driven methods is their detachment from clinical/theoretical relevance. Indeed, the unconstrained nature of these analytic pipelines allows them to detect any pattern that they can pull from the data, be it signal, noise, artifact, or spurious finding. To counter this, high-quality data-driven pipelines are now compelled to test the resultant model’s ability to generalize beyond the tested data. Best practice calls for the use of independent *training* and *testing* sets, which are comprised of completely separate samples of participants. Thus, a machine learning model will be trained to maximum predictiveness in training sample A, and then be tested for generalizability in testing sample B. This is a critical step within the machine learning process, to avoid overfitting, and to increase confidence in the reliability/validity of the extracted features. However, the reality is that this requires a sufficient number of participants to construct two independent samples, which is not always possible in psychiatric neuroscience, where access to patient populations and neuroimaging technology can be difficult. As such, other validation methods have been developed, including leave-one-out and k-fold cross-validation techniques, which use variations on bootstrapping procedures to afford validation within a single group of participants. While independent training/testing samples is the gold standard, the goal of each technique is the same: to minimize the likelihood that overfitting of the test data will occur, and to provide a critical demonstration of the potential utility of the model as a predictive device. At this point, some form of cross-validation in machine learning pipelines is essentially mandatory.

### 3.2. Multivariate approaches

The supervised/unsupervised models discussed above all focused on a specific neurobiological feature of interest. However, the computational capacity and enhanced signal/noise ratios of emerging machine learning methods also afford models that incorporate increasingly complex multimodal relationships. This is important, as most research suggests that multivariate models can outperform otherwise equivalent univariate models, with prediction classification rates at times beyond 90% (Kambeitz et al., 2017). One popular approach for combining multimodal biomarkers of disease states is joint ICA (jICA), which concatenates two or more modalities into a single data stream (Sui et al., 2011). Lottman et al. (2018), for instance, combined measures of gray matter, white matter, cerebrospinal fluid, and the amplitude of low-frequency fluctuations into a single jICA model, toward successful differentiation of first-episode schizophrenia patients and controls (Lottman et al., 2018). Similarly, Ouyang et al. (2015) combined both gray matter volume and white matter functional anisotropy profiles into a single jICA model, toward the identification of multimodal features that distinguished Alzheimer’s patients from controls. Combining modalities in this way affords the development of a more comprehensive model, capable of taking into account relationships and unique features of each brain metric. Several studies have employed multimodal neuroimaging pipelines toward the identification of neurobiological differentiators of antisociality (e.g., Steele et al., 2015) and SUDs (e.g., Seo et al., 2015; Whelan et al., 2014). However, the majority of this work, like its unimodal counterparts, has focused solely on identifying neurobiological features that can differentiate patient from control populations, rather than targeting issues of comorbidity/heterogeneity.

Of particular relevance for the present paper is the ability to also merge neuroimaging data with other modalities such as electrophysiological (e.g., Valdes-Sosa et al., 2009), phenotypic (Anderson et al., 2014), and genetic (e.g., Calhoun et al., 2009; Meda et al., 2012) data. Indeed, the process of mapping neurobiological features onto dimensional classifications of psychiatric disorders will almost certainly require a sophisticated merging of personality, clinical, and neurobiological data, to afford unique insights into the multivariate relationships between these constructs (e.g., Hilbert et al., 2017), and to help link biological predispositions to phenotypic characteristics (see Brazil et al., 2018). One particularly interesting method that has shown initial utility combines cross-modal data as simultaneous predictors of psychiatric categories, toward the development of individual phenotypes (i.e., merged neuropsychiatric “fingerprints”; Smitha et al., 2017). Evidence for this type of approach can be seen in Whelan et al. (2014) who successfully combined a wide variety of behavioural, cognitive, personality, environmental, and neuroimaging metrics into a single model to predict binge drinking in at-risk youth (see also Ding et al., 2017). Of relevance to personality-based classification systems like HiTOP, results from this study indicated that neural, environmental, and personality-based features each provided unique explanatory power within the resultant predictive model.

Other applications of multimodal pipelines have used techniques to evaluate for common elements between cross-modal metrics. Zhao et al. (2018), for instance, used unsupervised clustering methods to create neurobiological profiles of autism, ADHD, Alzheimer’s, and PTSD/post-concussive syndrome, and subsequently compared the relationship between these profiles and existing clinical and phenotypic metrics of these disorders. Interestingly, while there was a high degree of similarity between the clinical and connectivity metrics for autism and PTSD/post-concussive syndrome, the similarity was higher between phenotypic and connectivity clusters for Alzheimer’s and ADHD. Thus, this data provides evidence of a potential disconnect between existing clinical and underlying neurobiological metrics, and also highlights that this similarity/disconnect may vary importantly by disease/dysfunction type. The possibility that similar effects characterize different subclusters of externalizing disorders (e.g., SUD/ASPD/psychopathy or disinhibitory/antagonistic antisociality) may be of considerable import and remains almost entirely uninvestigated.

### 3.3. Latent factor approaches

Other emerging machine learning methods (e.g., latent factor approaches, hierarchical clustering, deep learning models) have been designed to allow for the modeling of complex hierarchical structures, such that specific lower-order features can be modeled as components of more general higher-order latent factors. Models of this sort can go beyond simple parsing of the neural response patterns between two disorders, and can instead consider the relationship between those disorders in a more sophisticated, ecologically valid manner. For instance, by allowing specific neurobiological characteristics to load simultaneously onto multiple clinical labels, latent factor models can assess the extent to which those characteristics are representative of one specific disorder, or to a broader category of disorders linked by a latent clinical factor. Cha et al. (2016), for instance, used a latent factor approach to delineate shared/unique neurobiological components of Major Depressive Disorder (MDD) and Generalized Anxiety Disorder (GAD; Cha et al., 2016), where comorbidity of symptom characteristics has long been acknowledged (Hamilton et al., 2014). Similar modelling of shared/unique variance has rarely been undertaken within the externalizing domain (but see more rudimentary approaches recently undertaken by Denomme et al., 2018; Denomme & Shane, 2020; Simard et al., 2021), but may be equally informative for distinguishing the extent to which specific neurobiological abnormalities relate to core underlying antisocial characteristics, to consequences of an antisocial/substance-abusing lifestyle, or to shared latent factors. Indeed, HiTOP currently conceptualizes externalizing as composed of two higher-order factors (disinhibited/antagonistic externalizing), and the triarchic model of psychopathy has conceptualized psychopathy as the higher-order factor of three subdimensions (boldness, meanness, and disinhibition). Within a biobehavioral framework, dimensions of this nature may provide neurobehaviorally-based traits that could serve as intermediary constructs to explain the relationship between psychopathological states (e.g., as conceptualized by HiTOP) and neurobiological underpinnings (e.g., as conceptualized by RDoC; see Perkins et al., 2019 for the elaboration of these ideas). It goes without saying that hierarchical frameworks of this nature will require the employ of hierarchical models to fully test their predictions; that only a handful of machine learning studies on the neurobiological underpinnings of SUD/antisociality have to date taken this approach marks the significant need for additional research.

Alternately, by allowing multiple symptom combinations to load on single disease categories (see Ruiz, Valera, Blanco, & Perez-Cruz, 2014), issues related to disorder heterogeneity can be increasingly targeted. For instance, some work suggests that SUDs may be conceptualized as occurring with or without withdrawal symptoms (e.g. Denomme & Shane, 2020). Whereas, DSM5’s crude approach classifies all SUD patients together so long as they show some combination of required symptoms, a hierarchical approach that can individually model the prevalence and relevance of each symptom can allow researchers to delve further into these more nuanced questions. Techniques of this nature have demonstrated utility in parallel fields. For instance, Tursich et al. (2015) identified distinct resting-state functional connectivity patterns that delineated hyperarousal and depersonalization components of PTSD (Tursich et al., 2015). Similarly, Drysdale et al. (2017) discovered novel neurophysiological subtypes of depression based on resting-state functional connectivity dynamics. As a result of such differentiation, specific research into course, prognosis, or treatment opportunities may ensue.

Furthermore, work of this nature may afford novel reclassification of disorder types, in line with RDoC and/or HiTOP initiatives. For instance, Van Dam et al. (2017) used factor analysis, in combination with hierarchical clustering, to group a 347-person community sample into “phenotypic communities” based on a combination of behavioral, psychiatric, and resting-state functional connectivity metrics. Techniques of this nature may have considerable significance for externalizing psychopathology, where heterogeneity remains high. For instance, psychopathy is commonly separated via factor analysis into two primary factors and/or four individual facets (interpersonal, affective, behavioral, criminogenic); however, the shared/unique neurobiological components underlying these factors/facets remain poorly understood (see Cohn et al., 2015 for a slightly different neurobiologically motivated delineation). Data-driven techniques that evaluate neurobiological underpinnings via hierarchical clustering, or other latent factor approaches, may achieve important insights into the relationships between these factors/facets, and the extent to which they show shared or unique underlying neurobiological features.

Finally, latent factor structures may facilitate the evaluation of more mechanistically-motivated questions about the relationships between cross-modal constructs (Friston, Redish, & Gordon, 2017; Meyer-Lindenberg, 2009; Zald & Lahey, 2017). To this end, neuroimaging metrics may themselves be conceptualized as “intermediate latent factors” within broader computational models that seek to model relationships between factors at different levels of analysis (see Brazil et al., 2018; Perkins, Latzman, & Patrick, 2019). While only a small amount of work of this nature has been undertaken in psychiatry to date (see Kircanski et al., 2018 as a valuable example), it is more prevalent in other fields – particularly within the Alzheimer’s literature where quite advanced work is being undertaken. In perhaps the most sophisticated of these approaches to date, Zhang, Marmino et al. (2016) used a Bayesian model to automatically identify distinct latent factors from atrophy patterns and cognitive deficits in late-onset Alzheimer’s disease (AD) dementia patients. These multimodal neural/cognitive latent factors were then used to predict distinct patterns of tau depositions. By placing these metrics as intermediary factors within data-driven models, work of this nature can go beyond searching for potential biomarkers and can instead delve for mechanistic insights into the underlying nature of the disorder. Work of this nature has not yet been pointed toward externalizing disorders, but may offer unique opportunities to differentiate mechanisms related to the antisocial personality, and to substance use/abuse, respectively. For example, as but two possibilities: (a) amygdala volume, connectivity, and sensitivity could be used to form a broader latent “amygdala” factor, which could itself load differentially on subtypes of antisociality; (b) approach and avoidance circuitry could be combined into a “drug-sensitivity” latent factor and used to predict response to drug cues in those with/without withdrawal symptoms.

## 4. Existing Work Employing ML Techniques Toward Antisociality and SUDs

To gain a more comprehensive sense of the state of the field to date, we conducted a meta-search for articles that employed machine learning approaches to predict either antisociality or substance use constructs via neuroimaging metrics. This search elicited 53 studies that met the selection criteria (see Table 1 for a list of these studies, and supplementary methods for all details regarding the meta-search pipeline employed). The majority of these studies have reported moderate-to-high success in predicting clinically relevant features, which speaks to the potential feasibility and utility of the approaches employed (though see Gowin et al., 2019). Nonetheless, work in this area remains nascent, and many opportunities to make full use of data-driven pipelines remain untapped. Below we provide a brief overview of the studies conducted to date, separated by their primary focus on either diagnosis/assessment, prognosis/course, heterogeneity/subtyping, or treatment/rehabilitation. To emphasize the critical importance of validation steps in machine learning pipelines, we discuss below only those studies that included an acceptable form of cross-validation (i.e., leave-one-out cross-validation, k-fold cross-validation, independent training/testing samples).

### 4.1. Diagnosis/assessment

Mirroring somewhat the broader neuropsychiatric literature (see Walter et al., 2019; Woo et al., 2017), the majority of work in the externalizing field to date (˜66%) has focused on aiding diagnosis/assessment of participants based on the presence/absence of clinically relevant features. In turn, all but one of these studies have employed supervised learning techniques, wherein target categories were provided *a priori* (e.g., DSM diagnostic categories). Classification accuracy has generally been moderate to high (e.g. ˜65–85%) regardless of the neuroimaging modality chosen, including the use of regional cerebral blood flow to predict methamphetamine dependence (Li et al., 2019); resting-state functional connectivity dynamics to predict smoking status (Pariyadath et al., 2014; Wetherill et al., 2018), cocaine dependence (Mete et al., 2016; Zilverstand et al., 2018), alcohol use disorder (Zhu et al., 2018) or ASPD diagnosis (Tang et al., 2013a); structural morphology to predict various SUD diagnoses (Mackey et al., 2019), psychopathic traits (Steele et al., 2017) or conduct disorder (Zhang et al., 2019, 2018); diffusor tensor imaging to predict smoking status (Zhao et al., 2019) or; resting-state activity to classify heroin dependence (Zhang et al., 2011), cocaine dependence (Sakoglu et al., 2019), or ASPD (Tang et al., 2013b). Because the vast majority of these studies have focused on a single modality, it is difficult to draw firm conclusions regarding the extent to which the similar classification accuracies suggest shared variance across modalities, or an upper limit on classification. That said, a handful of recent studies have incorporated multiple modalities into their predictive pipeline. In one such study, Ding et al. (2017) distinguished smokers from nonsmokers with 75% accuracy via multiple resting-state modalities. In the second study, Kamarajan et al. (2020) classified patients with alcohol use disorders with 76% accuracy via a combination of default mode activity, neurophysiological test scores, and impulsivity levels. Finally, Gowin et al., (2020) compared models with psychosocial metrics, with neuroimaging metrics, or a combined psychosocial/neuroimaging model. While the combined model did show the highest level of prediction (AUC = .86), it only barely outperformed he psychosocial model alone (AUC = .84). Thus, the extent to which multimodal models will aid diagnostic classification should remain a focus of attention (research from other domains does suggest that incorporation of multiple modalities can aid classification success (Kambeitz et al., 2017)).

Equally importantly, it should be noted that all studies to date have focused on a single disorder category and have sought only to distinguish forensic/psychiatric from healthy populations; thus, the ability to distinguish differential diagnoses, or to model comorbidity, remains almost entirely untested at present. Several studies focused on predicting ASPD diagnoses (Tang et al., 2013a; Tang et al., 2013b) or conduct disorder (Zhang et al., 2019, 2018) did exclude participants based on recent substance abuse, which affords some control over sample variance, and increases the likelihood that the neural features included in the model related to the ASPD/CD diagnoses. Nonetheless, exclusionary practices of this nature still negate the ability to test for comorbid/covariation effects; moreover, given how high substance abuse/conduct disorder comorbidity rates are, generalizability of reported effects may be a concern.

### 4.2. Prognosis/course

Our search identified 10 studies that have to date employed machine learning techniques to predict issues related to prognosis/course of externalizing disorders (8 focused on SUD; 2 on antisociality). Unlike most of the studies that have focused on diagnosis/assessment, work in this space has generally taken a quite sophisticated multivariate regressive approach, such that not only neuroimaging data, but also a variety of sociodemographic, personality, clinical, and/or neural predictor variables have been incorporated into a single multimodal predictive model (Clark et al., 2014; Bertocci et al., 2017; Squeglia et al. 2017; Kiehl et al., 2018; Whelan et al., 2014; Spechler et al., 2019). A major advantage of this approach is that it can maximize prediction capacity while also affording measurement of each predictor’s unique contribution to the model. However, in order to fully achieve this goal, regressive models must be handled carefully, with predictors stepped into the models in deliberate fashion – in contrast, the majority of studies to date have dumped all predictors into the model simultaneously to achieve the greatest overall prediction levels. In one particularly sophisticated study, Whelan et al. (2014) generated a multivariate model of current and future adolescent alcohol misuse (*n* = 692) to demonstrate that experiential, neurobiological, and personality features each served as unique and important antecedents of binge drinking. Similar approaches by other teams have also utilized multimodal neuropsychosocial models to successfully predict future substance use (Bertocci et al., 2017), future cannabis use (Spechler et al., 2019), and SUD relapse (Clark et al., 2014; Gowin et al., 2015). To repeat, however, these studies generally sought to obtain maximum prediction, rather than to isolate unique variance, thus limiting the ability to model individual predictor/outcome relationships.

More disappointing, none of the eight studies that focused on SUD incorporated any measure of antisociality in their pipeline. As a result, there is currently no way to handle considerations of comorbid relationships. The two studies focused on antisociality (Steele et al., 2015; Kiehl et al., 2018), in contrast, did include measures of substance use/abuse in their pipelines. However, the goal of these studies was again to achieve maximal specificity/sensitivity, and thus issues of comorbidity, or multicollinearity, were not well considered.

### 4.3. Heterogeneity/subtyping

Our search identified five studies (four focused on SUD and one focused on antisocial personality features) that have to date attempted to use machine learning architecture to aid subtyping of externalizing psychopathology. In two of these studies, subtyping was based on symptom severity, with supervised SVMs aiming to categorize individuals with more versus less severe symptom characteristics: Wetherill et al. (2018) attained 88% accuracy in predicting the severity of nicotine use disorder via within-network connectivity of various resting-state networks, while Steele et al. (2017) reported between 69%-80% prediction accuracy using structural MRI data to categorize adolescents into those with low/high psychopathic traits. Two additional studies used regression-based approaches to predict continuous variation in alcohol use severity (Fede et al., 2019), and years of cocaine use (Joseph et al., 2019), via resting-state functional connectivity metrics. Finally, one additional study, presented as a conference paper took a quite different approach that is worth expanding on here: Zilverstand et al. (2018) combined unsupervised methods with clustering techniques to identify cocaine-dependent and nondependent individuals who showed similar covariation patterns in their resting-state functional connectivity dynamics. Subsequently, the neurocognitive features of participants characterized by each of the resultant connectivity profiles were evaluated and shown to separate individuals with high reward sensitivity from those with low self-control traits. As the only study of its type within the externalizing domain, this study serves as a valuable example of how unsupervised methods can motivate novel reclassifications of clinical constructs, and how the use of neuroimaging metrics as intermediate latent factors can help bridge neurobiological features with behavioral/cognitive phenotypes.

### 4.4. Treatment/rehabilitation

Our search identified only four studies to date (all focused primarily on SUD) that have employed data-driven techniques to evaluate treatment/rehabilitation success in externalizing populations. The first constituted a rare PET study, which used SVM to demonstrate that resting-state D2/D3 binding potential in the nucleus accumbens could predict success in a contingency management program (built off of the community reinforcement treatment approach; Higgins and Budney, 1993) with an 82% accuracy rate (Luo et al., 2014). The second used a cross-validated logistic regression pipeline to predict 3-month relapse rates via functional activity during a drug/food cue reactivity task in a sample of veterans in a 28-day residential treatment program (MacNiven et al., 2018). The third and fourth studies (Yip et al., 2019; Lichenstein et al., 2019) both used a new technique – connectome predictive modeling (CPM) – to together demonstrate that different neural predictors were required to successfully predict cocaine and opioid abstinence rates throughout a 12-week treatment protocol. While the predictive success of these studies is encouraging, it must be noted that the sample sizes of these four studies (*n* = 25, *n* = 36, *n* = 53, and *n* = 53, respectively), and the diverse nature of neuroimaging metrics employed as predictors, can allow for only the most preliminary of conclusions at present.

The next closest study to evaluate treatment-related success was a functional connectivity study conducted by Steele and colleagues that predicted treatment completion (thus not necessarily success) in a larger sample of stimulant-/heroin abusers (*n* = 139; Steele et al., 2018); see also two similar EEG-based studies by the same group that falls outside the scope of this review; Fink et al., 2016; Steele et al., 2014). While both psychopathic traits and dependence status were included as continuous predictors in this study, as with other work from this group, the primary goal was to achieve maximum prediction levels, which led predictors to be included in ways that precluded full evaluation of shared/unique variance. Moreover, while important, work focused on treatment completion cannot speak directly to mechanisms underlying externalizing psychopathology; thus, future work focused more directly on treatment/rehabilitation success would be encouraged.

### 4.5. Recommendations for future work

#### 4.5.1. Collect data on both addiction and antisociality

Our search identified only a small number of studies that have considered neuroimaging metrics of both addiction and antisociality in the same machine learning pipeline. Moreover, as described above, because the goal of these studies was generally to achieve maximal prediction of disorder classification, models were not set up in a way to optimize insights into the potential shared/unique relationships between the identified neural markers and addiction/antisociality. We thus recommend that SUD/antisociality researchers include continuous metrics of both disorders (e.g., psychopathic traits; substance use severity) as standard practice, and that issues of comorbidity/covariation be explicitly evaluated. A small amount of work outside the machine learning literature has moved in this direction. For instance, Denomme et al., 2018 used fMRI to identify regions related to enhanced cue reactivity in cocaine-dependent individuals, and undertook subsequent regression analyses to dissect the extent to which activity within each of these regions related more closely to continuously derived metrics of psychopathic traits (i.e., PCL-R total score) or substance use severity (i.e., years of lifetime use; see also Denomme & Shane, 2020; Simard et al., 2021). And utilizing a quite distinct approach, Hyatt et al. (2019) created neuroanatomical profiles (based on their five-factor traits) on 1101 participants from the Human Connectome Project, and reported that while neuroanatomical profiles of Agreeableness and Conscientiousness showed medium-to-large relationships with externalizing psychopathology, neuroanatomical profiles of Agreeableness related more closely to metrics of antisocial behavior, while neuroanatomical profiles of Conscientiousness related more closely to metrics of substance abuse. A valuable next step for the field will be to utilize latent factor models capable of more fully incorporating shared/unique variance calculations, as well as issues related to comorbidity/heterogeneity.

#### 4.5.2. More work with dimensional variables

Most studies have tended to use supervised machine learning techniques (e.g., SVM) that match neuroimaging-based biomarkers to interview-based clinical diagnoses. While valuable, as reviewed above, these categories are themselves inaccurate proxies for the underlying disorder, created through clinical consensus to ensure diagnostic reliability, but not necessarily maximal validity (see Insel et al., 2010; Widiger, 1992). Referring back to these classifications thus poses an inadvertent constraint on any prediction algorithm – moreover, it ensures that data-driven neuroimaging analyses will not *improve upon* current diagnostic categories.

We thus recommend an increased focus on dimensional constructs that can allow for the identification of novel relationships and new classification strategies (see as example Yip et al., 2018). One way to target issues of comorbidity within a data-driven pipeline would be to combine an algorithmic clustering approach (e.g., ICA) with hierarchical regression, to evaluate the extent to which ICA-extracted neural components load predominantly on ASPD, on SUD, or on a latent externalizing factor. Alternately, employing machine learning techniques that afford prediction of continuous outcomes (e.g., SVR; CPM) would allow researchers to take full advantage of the continuous nature of neuroimaging/psychiatric data, and to evaluate more sophisticated questions regarding the nature of externalizing psychopathology (e.g., disorder severity; treatment success).

#### 4.5.3. Considering neuroimaging markers as latent intermediate factors

HiTOP (and to some extent RDoC) encourage consideration of psychological dysfunction within a hierarchical framework; however, the majority of machine learning work to date has failed to take advantage of this hierarchical structure. Zilverstand et al. (2018) is an early exception, as this study incorporated an unsupervised machine learning pipeline to identify neural patterns that were subsequently related not to disorder categories, but rather to dimensional metrics of potentially related neurocognitive functions. This intermediary step of linking neurobiological features to underlying behavior/personality/cognition may be an integral step toward a true mechanistic understanding of disease pathology (Zhao et al., 2018). For instance, if a given neurobiological feature results from substance-induced exogenous factors, then we may anticipate relationships with metrics of substance use severity; if instead a given neurobiological feature results from underlying antisocial characteristics, then we may anticipate relationships with stable personality traits. Alternately, certain features may indeed relate equally to both disorders, and instead map onto a latent externalizing factor. Future work capable of constructing latent factor models will have the ability to interrogate questions of this nature, toward a more detailed understanding of the mechanisms underlying externalizing pathology.

### 4.6. The challenges ahead

#### 4.6.1. Small sample sizes

A common limitation of most studies to date is their reliance on small sample sizes, which reduces precision (or, in the parlance of machine learning, increases the likelihood of overfitting). Fortunately, with an increase in multisite and consortium efforts, large comprehensive datasets of this nature are becoming increasingly available. Several large studies have recently been reported (Espinoza et al., 2019), and consortium efforts are quickly organizing (e.g., ENIGMA; Thompson, 2019). These are perfect for the application of machine learning tools, and offer the prospects of sufficiently sophisticated analyses, within a reasonable time frame, to make the fine-grained distinctions necessary to answer critical questions pertaining to comorbidity/heterogeneity. Additional use of machine learning tools on these large datasets may increase the ability to predict prognosis and course of externalizing disorders by taking into account subject-specific factors (see (Walter et al., 2019) for elaboration), to afford early detection/identification of individuals with poor prognosis, or to afford new avenues for patient stratification and early intervention. Issues regarding sample size will continue to be a concern, however; particularly as deep-learning models gain additional traction in translational applications (for review, see Vieira et al., 2017), as these models may require sample sizes orders of magnitude larger than traditional ML pipelines.

#### 4.6.2. Expertise is scarce

Personality theory, psychiatry, neuroscience, and machine learning require quite diverse forms of expertise, which puts necessary limits on full integration of these disciplines. Thus, to large extent, the initiation of sophisticated machine learning architecture will require collaborative efforts that cut across disciplinary lines. In addition, and fortunately, recent advances in both software and hardware are making computational modelling and machine learning more accessible. For instance, recent multidisciplinary initiatives have sought to develop more user-friendly versions of computational (e.g., hBayesDM in R), and machine learning (e.g., EasyML; Hendricks & Ahn, 2017) software that can allow those familiar with theoretical, if not technical, requirements of these methods to develop sophisticated models (see Table 2 for a list of available packages). These advances will no doubt continue to increase accessibility as time goes on (see also (Durstewitz et al., 2019) for additional considerations of potential solutions.

**Table 1.** Summary of studies employing machine learning techniques toward evaluation of psychopathy/ASPD and/or substance use disorders

Study	S/U	ML Technique	Modality investigated	CV Technique	Training Set	Independent Testing Set	Outcome Variable	Prediction Accuracy(PA)/Other findings
*Diagnosis/Assessment*								
Amen et al., 2017	S	LDA	SPECT – rest and concentration task	LOOCV	982 CanUD 92 HC	–	CanUD versus HC	Rest: 92% PA Task: 90% PA
Chen et al., 2015	S	LDA	Resting-state regional homogeneity	LOOCV	29 violent juvenile offenders 28 HC	–	Juvenile violent offenders versus controls	89.5% PA
Chung et al., 2018	S	SVM	fMRI – stop signal task	LOOCV	40 CD 27 HC	–	CD versus HC	55–66% PA
Cope et al., 2014	S	SVM	VBM	LOOCV	20 homicide offenders 135 non-homicide offenders	–	Homicide versus non-homicide offenders	81% PA
Ding et al., 2015	S	SVM	VBM	10-fold CV and Independent sample	60 smokers 60 nonsmokers	28 smokers 28 nonsmokers	Smokers versus. nonsmokers	Training: 69.6% PA Testing: 64% PA
Ding et al., 2017	S	SVM	Multimodal rs-Fmri	10-fold CV	100 smokers 100 non-smokers	–	Smokers versus control	70.5–75.5% PA
Elton et al., 2014	S	LDA	Stop-signal fMRI	LOOCV	26 CD 18 HC	–	CD versus control	89.5% PA
Gowin et al., 2020	S	RF	Multimodal neuropsychosocial	10-fold CV	133 binge drinkers 252 nonbinge drinkers	44 binge drinkers 77 nonbinge drinkers	Binge versus nonbinge	AUC = .64
Guggenmos et al., 2020	S	WeiRD/ SVM	Multimodal neuroimaging	LOOCV	119 AD 97 HC	–	AD versus HC	˜73%-79% PA
Guggenmos et al., 2018	S	WeiRD/SVM	GM volume	LOOCV	119 AD 97 HC	–	AD versus HC	˜70–74% PA
Kamarajan et al., 2020	S	RF	rs-FNC	Bootstrapped validation	30 AUD 30 HC	–	AUD versus HC	76.67% PA
Li et al., 2019	S	SVM	ASL-CBF	Semi-independent sample	45 MD 45 HC	36 MD same 45 HC	MD/HD versus. HC	89% PA
Luo et al., 2020	S	SVM	Rs-ALFF	10-fold CV	51 HD 40 HC	–	HD versus HC	˜64% PA
Mackey et al., 2019	S	SVM	GM volume	Split-half CV	2140 SUD 1100 HC	–	Various SUD versus HC	AUC = .43−.78
Mete et al., 2016	S	SVM	SPECT-CBF	LOOCV and 10-fold CV	93 CD 69 HC	–	CD versus. Control	88% PA
Pariyadath et al., 2014	S	SVM	rs-FNC	LOOCV	21 smokers 21 nonsmokers	–	Smokers versus. nonsmokers	78% PA
Park et al., 2018	S	GAM	sMRI DTI	2-fold CV	34 moderate/heavy drinkers 671 no/low drinkers	–	Regular versus minimal drinker	57.4–76.5% PA
Ruan et al., 2019	S	SVM	rs-FNC SNP	Independent sample	95 drinkers 44 nondrinkers	52 drinkers 21 nondrinkers	Drinker versus nondrinker	Training: 86.7% PA Testing: 71.2% PA
Sakoglu et al., 2019	S	SVM	rs-FNC	LOOCV	58 CD 25 HC	–	CD versus control	81–95% PA
Sato et al., 2011	S	SVM, MLDA	VBM	LOOCV	15 PCL-R > 30 15 PCL-R < 30	–	PCL-R > 30 versus. < 30	80% PA
Squeglia et al., 2017	S	RF	fMRI – working memory/sMRI	Bootstrapped validation	137 adolescents	–	Moderate/heavy alcohol use versus. nonusers	74% PA
Steele et al., 2017	S	SVM	VBM	LOOCV	143 offenders 21 HC		High/low psychopathic traits versus. HC	69–82% PA
Tang et al., 2013a	S	SVM	rs-FNC	LOOCV	32 ASPD 35 HC	–	ASPD versus. HC	86% PA
Tang et al., 2013b	S	SVM	Regional homogeneity	LOOCV	32 ASPD 34 HC	–	ASPD versus HC	70% PA
Wang et al., 2018	U	Conv3d deep learning	sMRI	4-fold CV	61 smokers 66 nonsmokers	–	Smokers versus nonsmokers	80.6–93.5% PA
Wei et al., 2021	S	LASSO regression	rs-FNC	LOOCV	24 CD 24 HC	–	Empathy scores	Model explained 29.16% of the variance in empathy scores.
Wetherill et al., 2018	S	SVM	rs-FNC	10-fold CV	108 NUD 108 HC	–	NUD versus. control	88.1% PA
Yu et al., 2011	S	SVM	VBM	LOOCV	16 smokers 16 HC	–	Smokers versus HC	81.25% PA
Zhang et al., 2019	S	SVM	3D sMRI	LOOCV	60 Conduct Dis. 60 HC	–	Conduct Dis. versus. control	83% PA
Zhang et al., 2018	S	SVM, LR, RF	VBM	5-fold CV	60 Conduct Dis. 60 HC	–	Conduct Dis. versus. control	77.9%–80.4% PA
Zhang et al., 2016	S	SVM	rs-FNC	LOOCV	100 CD 100 HC	–	CD versus HC	72% PA
Zhang et al., 2011	S	SVM	rs-fMRI	LOOCV	12 HD 13 HC	–	HD versus. control	65% PA
Zhao et al., 2020	S	SVM	DTI	10-fold CV	70 smokers 70 nonsmokers	–	Smokers versus. nonsmokers	88.6% PA
Zhu et al., 2018	S	RF	Within-/between-network rs-FNC	Bootstrapped validation	46 AUD 46 HC	–	AUD versus HC	Within-network FNC: 87% PA Between-network FNC: 67.4% PA
*Prognosis/Course*								
Bertocci et al., 2017	S	LASSO regression	fMRI – reward and CT	10-fold CV	73 youth	–	2-year future substance use	83.6% PA
Clark et al., 2014	S	Multiple	Selective attention fMRI	10-fold CV	45 SUD	–	Relapse versus no relapse	77.8–89.9% PA
Kiehl et al., 2018	S	Cox regression	sMRI	10-fold CV	93 offenders	–	Reoffending	not reported
Gowin et al., 2019	S	RF	fMRI – reward related	IS	63 MUD	29 CUD	Relapse versus. abstinence	Training: 65% PA Testing: *NS*
Gowin et al., 2015	S	RF	fMRI – reward related	Bootstrapped validation	69 MUD	–	Relapse versus. abstinence	72–75% PA
Sekutowicz et al., 2019	S	SVM	Pavlonian-to-instrumental fMRI	LOOCV	52 detoxified AD	–	12-month relapse versus. abstinence	71.2% PA
Seo et al. 2015	S	SVM, NB, VQ	VBM/fMRI – cue reactivity	LOOCV	46 AD	–	3-month relapse versus abstinence	73–79% PA
Spechler et al., 2019	S	Logistic regression	Task-realted fMRI/sMRI	10-fold CV	1581 14 year olds	–	Cannabis/alcohol use	AUC = 0.65–0.82
Steele et al. 2015	S	SVM	fMRI/EEG – Go/No-Go	LOOCV	45 offenders	–	Reoffending	83% PA
Whelan et al., 2014	S	LG, Elasticnet	Multimodal fMRI/genetic/personality/environmental	10-fold CV	692 adolescents	–	Binge drinking	93–95% PA
*Subtyping/Heterogeneity*								
Fede et al., 2019	S	RF regression	Multimodal rs-FNC	10-fold CV and IS	59 with moderate/heavy alcohol use	24 with moderate/heavy alcohol use	Alcohol use severity	Training: R^2^: 98.7 Testing: R^2^: 33.2
Joseph et al., 2019	S	ALM	rs-FNC	10-fold CV	83 CUD	–	Years of cocaine use	*R* ^2^ = 8–20%
Steele et al., 2017	S	SVM	VBM	LOOCV	143 offenders 21 HC	–	High/Low PCL-YV	69.23% PA
Wetherill et al., 2019	S	SVM	rs-FNC	10-fold CV	108 NUD 108 HC	–	NUD versus. HC	88.1% PA
Zilverstand et al. 2018	U	K-centroid clustering	rs-FNC	LOOCV	42 CUD 32 HC	–	Within CUD heterogeneity	CUD subtypes classified via neurocognitive profile
*Treatment/Rehabilitation*								
Luo et al., 2014	S	SVM	rs-PET	10-fold CV	25 CUD	–	Treatment responders versus Nonresponders	82% PA
MacNiven et al., 2018	S	Logistic regression	fMRI – Cue reactivity	LOOCV	36 SUD 40 HC	–	Relapse versus abstain	75.8% PA
Steele et al., 2018	S	SVM	Task-FNC Go/No-Go	10-fold	139 offenders	–	Treatment completion versus dropout	80.6% PA
Yip et al., 2019	S	CPM	rs-FNC	LOOCV and independent Sample	53 CUD	45 CUD	Abstinence during treatment	Testing: 64–71% PA

*ML techniques:* SVM, Support vector machine; LASSO, Least absolute shrinkage and selection operator; LDA, linear discriminant analysis; MVPA, Multivariant/voxel pattern analysis; SVR, Support vector regression. *CV techniques:* CV, cross-validation; LOOCV, leave-one-out cross-validation. *Modality:* ASL, Arterial spin labeling; CBF, Cerebral blood flow; DTI, Diffusion tensor imaging; FNC, Functional connectivity; GM, Gray matter; rs, Resting-state; SNP, Single-nucleotide polymorphism; VBM, Voxel-based morphometry. *Population/Measure:* AD, alcohol dependence; ASPD, Antisocial personality disorder; AUD, Alcohol use disorder; CanUD, Cannabis use disorder; CD, cocaine dependence; CUD, Cocaine use disorder; HC, Healthy controls; HUD, Heroin use disorder; MUD, Methamphetamine use disorder; NUD, Nicotine use disorder; PCL-R, Psychopathy Checklist – Revised (Hare, 1991); PCL-YV = Psychopathy Checklist Youth Version (Forth et al., 2003); SUD, Substance use disorder.

**Table 2.** Some of the major open-access ML tools that include GUI interfaces (or have lower coding requirements)

*Tools with GUI interfaces*		
PRoNTo	Schrouff et al. (2013)	MATLAB toolbox with GUI interface (and MATLAB batch script creation capabilities), for a wide range of categorical and continuous ML methods.
Weka	Frank et al. (2016)	A general open-source machine learning software platform that can be accessed through a graphical user interface, standard terminal applications, or a Java API.
MALINI	Lanka et al. (2020)	A MATLAB toolbox for aiding clinical diagnostics using resting-state fMRI data.
GraphVar	Waller et al. (2018)	A user-friendly toolbox for comprehensive graph analyses of functional brain connectivity.
MANIA	Grotegerd et al. (2014)	A MATLAB-based software toolbox enabling easy pattern classification of neuroimaging data and offering a broad assortment of machine learning algorithms and feature selection methods. Between groups classifications are the main scope of this software, for instance, the differentiation between patients and controls.
MVPANI	Peng et al. (2020)	Multimodal, multivariate MVPA with a friendly graphical interface.
WekaDeepLearning4J	Lang et al. (2019)	A deep learning module for Weka that supports a variety of hierarchical and network-based models.
***Tools with lower coding requirements***		
Scikit-learn	Pedregosa et al. (2011)	Python module integrating a wide range of state-of-the-art machine learning algorithms for medium-scale supervised and unsupervised problems.
SHOGUN	Sonnenburg et al. (2010)	Multi-platform open-source machine learning library that offers a wide range of efficient and unified machine learning methods. Large cookbook of codes are available.
CosmoMVPA	Oosterhof et al. (2016)	A multivariate, multimodal (fMRI volumetric, fMRI surface-based, and MEEG) MATLAB toolbox.

#### 4.6.3. The black box

A common push/pull in analytic circles is the debate over data-driven versus theory-driven models. In this regard, data-driven methods are often criticized for their “black box” nature (Davatzikos, 2019), and for their disconnect from clinical and theoretical relevance. However, as Huys et al. (2016) point out, data-driven and theory-driven computational methods can complement each other, toward a deeper understanding of disorder characteristics. Common usage today is for data-driven methods to be employed to initially reduce the dimensionality of complex, multimodal datasets, and to allow for the construction of more digestible theoretical models (Allen et al., 2011). As the field is in its early days, this approach may offer the most efficient and productive ability to scour the massive amount of data that translational neuroimaging can provide. However, as the field matures, the opposite order may become increasingly beneficial: that is, theory-driven dimensionality reduction, followed by more powerful data-driven approaches that search through the theoretically relevant variables with the most sophisticated and efficient pattern analysis techniques (e.g., (Maia, Huys, & Frank, 2017)). This can retain a tight clinical/theoretical focus capable of supporting experimentally driven univariate analyses, while also achieving the analytical brawn that machine learning techniques can offer.

#### 4.6.4. Differential reliability of neurobiological predictors

Finally, while the paper’s primary intent is to highlight the strengths of using data-driven techniques to interrogate complex relationships between antisociality and SUD, we conclude by reminding that the validity and reliability of a predictive model can only be as high as the validity and reliability of the predictors used toward model prediction (see Fröhner et al., 2019). In this regard, the greatest strengths of these data-driven models may also be their greatest weakness: they will essentially move mountains to identify underlying patterns in the data - valid, reliable, or otherwise. Thus, as the use of machine learning models becomes more prevalent, researchers will need to be increasingly vigilant about their choice of predictor constructs, and of those predictors’ inherent reliability/validity. In this vein, it may be prudent to note that functional MRI, in particular, has been criticized recently for potentially low internal consistency (Infantolino et al., 2018) and test–retest reliability (Elliott et al., 2020). Though this issue is hardly unique to fMRI, or even neuroimaging (e.g., Anvari & Lakens, 2018; Świątkowski & Dompnier, 2017), and may indeed be somewhat overblown (Pannunzi et al., 2017), it does underline the need for best practices in the field: always carefully consider your model parameters, always aim to demonstrate clinical utility, and always make use of validation techniques to maximize the potential generalizability of study findings.
